# Intra-Operative Predictors of difficult cholecystectomy and Conversion to Open Cholecystectomy – A New Scoring System

**DOI:** 10.12669/pjms.341.13302

**Published:** 2018

**Authors:** Nauman Ahmed, Maaz ul Hassan, Maham Tahira, Abdul Samad, Hamad Naeem Rana

**Affiliations:** 1 Nauman Ahmed, Consultant General & Laproscopic Surgeon, Surgical Unit II, Shalamar Institute of Health Sciences, Lahore, Pakistan; 2 Maaz ul Hassan, Assistant Professor, Surgical Unit II, Shalamar Institute of Health Sciences, Lahore, Pakistan; 3 Maham Tahira, Medical Officer, Surgical Unit II, Shalamar Institute of Health Sciences, Lahore, Pakistan; 4 Abdul Samad, Shaheen Research Group, Karachi, Pakistan; 5 Hamad Naeem Rana, Professor of Surgery, Surgical Unit II, Shalamar Institute of Health Sciences, Lahore, Pakistan

**Keywords:** Conversion, Intra-operative, Laparoscopic cholecystectomy, Open surgery, Scoring system

## Abstract

**Objective:**

To evaluate the intra-operative scoring system to predict difficult cholecystectomy and conversion to open surgery.

**Methods:**

This descriptive study was conducted from March 2016 to August, 2016 in the Department of Surgery, Shalimar Hospital. The study recruited 120 patients of either gender, age greater than 18 years and indicated for laparoscopic cholecystectomy (LC). Intra-operatively all patients were evaluated using the new scoring system. The scoring system included five aspects; appearance and adhesion of Gall Bladder (GB), distension or contracture degree of GB, ease in access, local or septic complications, and time required for cystic artery and duct identification. The scoring system ranges from 0 to 10, classified as score of <2 being considered easy, 2 to 4 moderate, 5–7 very difficult, and 8 to 10, extreme. Patient demographic data (i.e. age, gender), co-morbidities, intra-operative scores using the scoring system and conversion to open were recorded. The data was analysed using statistical analysis software SPSS (IBM).

**Results:**

Among one hundred and twenty participants, sixty seven percent were females and the mean age (years) was 43.05 ± 14.16. Co-morbidities were present in twenty percent patients with eleven diagnosed with diabetes, six with hypertension and five with both hypertension and diabetes. The conversion rate to open surgery was 6.7%. The overall mean intra-operative scores were 3.52 ± 2.23; however significant difference was seen in mean operative score of converted to open and those not converted to open (8.00 ± 0.92 Vs. 3.20 V 1.92; p-value = 0.001). Among eight cases converted to open, three (37.5%) were in very difficult category while five (62.5%) were in extreme category. Moreover, age greater than 40 years and being diabetic were also the risk factors for conversion to open surgery.

**Conclusion:**

The new intra-operative scoring system is a valuable assessment tool to predict difficult laparoscopic cholecystectomy and conversion parameters to open surgery and its utility could improve patient's clinical outcome indicated for laparoscopic cholecystectomy.

## INTRODUCTION

Acute cholecystitis is a common complication of gall stone disease with reported incidence of 20% in literature.^1^ The pathophysiology is secondary bacterial inflammation of the gall bladder as a consequence of the cystic duct obstruction.^2^ Cholecystectomy is the commonest operation of the billiary tract and Laparoscopic cholecystectomy (LC) is the standard operative procedure for the treatment of symptomatic gallbladder disease.^3^,^4^ The need for conversion is neither a failure nor a complication but simply a step taken to ensure patient safety and avoid complications.^5^ LC is beneficial compared to traditional open cholecystectomy in terms of reduced pain postoperatively, decrease length of hospital stay, and improved and fast recovery of patients.^6^ Sometime, the laparoscopic cholecystectomy may pose undue difficulties during access or dissection and it is considered as a “difficult” when safe completion of the laparoscopic procedure cannot be ensured.^7^,^8^

There are numerous preoperative scoring systems proposing preoperative parameters reported for difficult cholecystectomy^9^,^10^. However there is no operative classification for laparoscopic surgery. Recently, operative grading system for laparoscopic cholecystectomy – a new scoring system, being the first “Operative classifications” was proposed by Surgrue M et al., (2015)^11^ to classify the difficult Cholecystectomy from mild to extreme on the basis of intraoperative predicators. The present study was conducted to help us to identify how valid the proposed intra-operative scoring system is to predict conversion of LC to open surgery.

## METHODS

This descriptive cross sectional survey was conducted at Department of Surgery, Shalimar Hospital for six months from March 2016 till August 2016. Patients greater than 18 years of age of either gender, presenting with symptomatic gallstones and indicated for laparoscopic cholecystectomy were included in this study. The exclusion criteria were jaundice, malignancy and patients infected with hepatitis B or C. Pre-operative assessment was performed and Standard four port technique was used for LC.

Structured questionnaire was used to record the findings. Demographic data (i.e. age, gender), anthropometric measurements (i.e. weight, height and body mass index), co-morbidities (i.e. diabetes and hypertension) were recorded. Moreover, all patients were evaluated using the new scoring system.^11^ The scoring system included five aspects; appearance and adhesion of Gall Bladder (GB), distension or contracture degree of GB, ease in access, local or septic complications, and time required for cystic artery and duct identification. The new operative scoring system is described in Table-I. The scoring system ranges from 0 to 10, classified as score of less than 2 *(easy)*, 2 to 4 *(moderate)*, 5–7 *(very difficult)*, and 8 to 10 *(extreme)*. Importantly, LC converted to Open was recorded.

**Table-I:** Operative Grading System for Cholecystitis Severity.

Operative Grading System	Score
*Gallbladder appearance*	
No Adhesions	0
Adhesions < 50% of GB	1
Adhesions burying GB	3
Maximum	3
*Distension/Contraction*	
Distended Gall bladder or Contracted shrivelled GB	1
Unable to grasp with atraumatic laparoscopic forceps	1
Stone ≥1 cm impacted in Hartman's Pouch	1
*Access*	
BMI >30	1
Adhesions previous surgery limiting access	1
*Severe Sepsis/Complications*	
Bile or Pus outside GB	1
Time to identify cystic artery and duct >90 minutes	1
Total Maximum	10

The study was conducted after the approval from the ethical review committee of Shalimar Hospital. Written informed consent was taken prior to recruitment of participants in the study. Study participants were completely briefed about the procedures of laparoscopic cholecystectomy, possible complications and possibility of conversion of LC to open procedure, purpose of the research, benefits and harms associated with the research. Anonymity and confidentiality of the study participants were maintained throughout the research.

### Data Analysis

Data was entered and analyzed using SPSS version 21 (IBM). Once the data was entered in the analytical software it was weighted twice for incorrect entries. Qualitative or categorical data (i.e. gender, age categories, co-morbidities, conversion to open etc.) were presented as frequency and percentage while quantitative data (i.e. age, weight, BMI and scores of intra operative scoring system) was presented as mean ± standard deviation. The mean scores of new intra operative scoring system were compared in two independent groups (converted to open as yes or no) using the independent t test. However, the categorical variables were compared in the two groups using chi square statistics. Where the assumptions of chi square were not satisfied Fisher exact test was used. For the inferential statistics p-value < 0.05 was considered significant.

## RESULTS

The present study enrolled one hundred and twenty participants indicated for laparoscopic cholecystectomy. The mean (SD) age in years of the study participant was 43.05 (14.16) years. Around fifty percent of the participants were greater than forty years. Moreover, a greater proportion of females (55%) were recruited in this study. Importantly, the body mass index of the study participants enrolled was higher with mean (SD) a 26.53 (5.18) Kg/m^2^. Furthermore, no co-morbidity was reported in more than eighty percent of the participants enrolled. Around eighteen percent of participants having diagnosed with co-morbidity, six (5%) were hypertensive, eleven (9.2%) were diabetic and five (4.2%) were both diabetic and hypertensive. Details of the characteristics of the study participants are given in Table-II.

**Table-II:** Characteristics of the study participants.

Characteristics	n (%) or Mean ± SD
Age (years)	43.05 ± 14.16
Age Categories	
≤ 40 years	59 (49.2%)
> 40 years	61 (50.8%)
Gender	
Male	54 (45%)
Female	66 (55%)
Weight (Kg)	69.14 ± 16.70
Body Mass Index (Kg/m^2^)	26.53 ± 5.18
Co-morbidities	
No	98 (81.7)
Hypertension	6 (5)
Diabetes	11 (9.2)
Hypertension and Diabetes	5 (4.2)

Among, one hundred and twenty participants recruited in this study, eight patients (6.7%) were converted to open surgery. Details of the comparison of intra-operative finding using the new scoring classification in patients indicated for laparoscopic surgery converted to open and those who did not are shown in Table-III. Significant difference was found in gall bladder appearance. All eight patients converted to open had adhesions burying gall bladder compared to around twenty four percent not converted to open (p-value < 0.001). Moreover, significantly high proportion of patients had distended/ contracted gall bladder (100% Vs. 38.4%; p-value = 0.025), unable to grasp (100% Vs. 53.6%; p-value = 0.009), stone greater than or equal to 1 cm impacted in Hartman's pouch (62.5% Vs. 23.2%; p-value = 0.027), bile and pus outside gall bladder (100% Vs. 24.1%; p-value < 0.001) and time required for cystic artery and duct identification greater than 90 minutes (75% Vs. 9.8%; p-value < 0.001) in converted to open and not converted to open group (Table-III).

**Table-III:** Comparison of Intra-operative findings in patients converted to Open and those not converted to Open Cholecystectomy using New Scoring System.

Intra Operative Findings	Converted to Open (n = 8)	Not Converted to Open (n = 112)	Total (n = 120)	P-value
Gallbladder appearance				
No Adhesions	0 (0)	49 (43.8)	49 (40.8)	0.001
Adhesions < 50% of GB	0 (0)	36 (32.1)	36 (30)	
Adhesions burying GB	8 (100)	27 (24.1)	35 (29.2)	
Distension/Contraction				
*Distended/ Contracted GB*				
Yes	8 (100)	69 (61.6)	77 (64.2)	0.025
No	0 (0)	43 (38.4)	43 (35.8)	
Unable to grasp				
Yes	8 (100)	60 (53.6)	68 (56.7)	0.009
No	0 (0)	52 (46.4)	52 (43.3)	
Stone ≥1 cm impacted in Hartman's Pouch				
Yes	5 (62.5)	26 (23.2)	31 (25.8)	0.027
No	3 (37.5)	86 (76.8)	89 (74.2)	
Access				
BMI >30				
Yes	3 (37.5)	26 (23.2)	91 (75.8)	0.297
No	5 (62.5)	86 (76.8)	29 (24.2)	
Adhesions previous surgery				
Yes	2 (25)	22 (19.6)	24 (20)	0.502
No	6 (75)	90 (80.4)	96 (80)	
*Severe Sepsis/Complications*				
*Bile or Pus outside GB*				
Yes	8 (100)	27 (24.1)	35 (29.2)	0.001
No	0 (0)	85 (75.9)	85 (70.8)	
*Time to identify cystic artery and duct >90 minutes*				
Yes	6 (75)	11 (9.8)	17 (14.2)	0.001
No	2 (25)	101 (90.2)	103 (85.8)	

Details of the intra-operative scores and categories classification of the patients enrolled using the new scoring system are shown in Table-IV. The overall mean (SD) of new intra-operative scoring classification was 3.52 (2.23). Majority, fifty nine patients (49.2%) lied in moderate, followed by very difficult (25.8%), mild (20%) and extreme (5%) category. Significant difference lied in intra operative scores with patients having converted to open had higher mean score of 8 compared to those not converted having mean score of 3.2. Moreover, significantly high proportion of patients in extreme category were converted to open (62.5% Vs. 0.9%; p-value = 0.001).

**Table-IV:** Comparison of Intraoperative Scores/ Categories of the patients converted to Open and those not converted to Open Cholecystectomy using New Scoring System.

Intra Operative Scores/ Categories	Converted to Open (n = 8)	Not Converted to Open (n = 112)	Total (n = 120)	P-value
Scores	8.00 ± 0.92	3.20 ± 1.92	3.52 ± 2.23	0.001
*Categories*				
Less than 2 (Mild)	0 (0)	24 (21.4)	24 (20)	0.001
2 – 4 (Moderate)	0 (0)	59 (52.7)	59 (49.2)	
5 – 7 (Very difficult)	3 (37.5)	28 (25)	31 (25.8)	
8 – 10 (Extreme)	5 (62.5)	1 (0.9)	6 (5)	

Moreover, significant difference lied in patient's age and co-morbidities (i.e. diabetic) in patients indicated to laparoscopic cholecystectomy converted to open or not. Finally, greater proportion of patients aged greater than 40 years (75%) were converted to open compared less than fifty percent in the similar age category was having converted to open surgery. Moreover, around thirty seven percent patients having diabetes were converted to open, while around seven percent of diabetic patients were not converted to open.

## DISCUSSION

The results of the present study highlighted that patients indicated for laparoscopic cholecystectomy having significantly higher mean operative scores were more likely to be converted to open surgery. Moreover, within the operative scoring system, significantly higher proportion of patients with adhesion burying the gall bladder, distended gall bladder, unable to grasp, Stone ≥1 cm impacted in Hartman's Pouch, bile or pus outside gall bladder and time to identify cystic artery and duct > 90 minutes were converted to open. Finally, greater proportion of patients aged greater than 40 years (75%) and around thirty seven percent patients having diabetes were converted to open.

In the present study conducted, among, one hundred and twenty patients, slightly less than seven percent were converted to open surgery. The recent studies conducted have reported the conversion rates varying in the wide range of 1.5–19%.^12^,^13^ In developed health setting the conversion rate has been reportedly low as compared to less developed healthcare setting of developing countries. This may be due to the ease of availability of most modern and latest laparoscopes and improved training and skills of surgeons in the developed countries as against less developed countries. Studies from Pakistan has reported the conversion rate as 8.7%, 3.5% and 6.5%.^14^-^16^

Significant differences were found in intra operative scores with patients having converted to open had higher mean score of 8 compared to those not converted having mean score of 3.2. Moreover, significantly high proportion of patients (62%) in extreme category was converted to open. The operative scoring system is good enough for the prediction of conversion to open surgery. The study by Vivek MA et al.^17^ reported scoring assessment of difficulty in more than three hundred patients who underwent LC and found the scoring assessment precise and valuable for predicting the difficulty in procedure and early identification of need for conversion. However, the grading system is quite complex as using twenty two parameters that also included four intra-operative parameters (distended/contracted or inflamed GB, hanging liver edge, and cirrhosis). Gupta validating another scoring system proposed by Randhawa and colleagues^18^ reported less operative features, only an ultrasonographically thickened (≥4 mm) GB wall, and an impacted stone.^19^ Thus, the proposed new operative scoring system is good enough being the first to outline key operative findings at laparoscopic cholecystectomy but its validity needs to be tested in future large prospective series before potentially serving as validated scoring system.

### Limitations of the Study.

Firstly, the percentage adhesion of gall bladder was subjectively assessed. Secondly, the study had limited sample size of only one hundred and twenty patients being recruited from a single study site. Considering the smaller sample binary logistic regression with outcome as conversion to open (yes/ no) was not performed. The results of the binary logistic regression would have provided the valuable clinical information of independent role of each of the risk factors. Thus, in future multicentre study with adequate sample size should be conducted to identify the validity and predictive capability of intra-operative scoring system for conversion to open surgery.

## CONCLUSION

The findings of the present study showed that new intra-operative scoring system is a valuable assessment tool to predict conversion to open surgery and its utility could improve patient's clinical outcome indicated for laparoscopic cholecystectomy. Using, this new operative scoring system, surgeons could better predict operatively cases which will likely be converted to open. The classification could be extremely beneficial in improving patient's outcome.

### Authors' Contribution

**NA & HNR** conceived, designed and did statistical analysis & editing of manuscript.

**MH and MT** assisted in literature review, data collection and manuscript writing.

**AS** did literature review, statistical analysis, manuscript writing and editing of manuscript.

**NA** takes the responsibility and is accountable for all aspects of the work in ensuring that questions related to the accuracy or integrity of any part of the work are appropriately investigated and resolved.
